# An open‐label, single‐arm, dose‐escalating concentration–QT study to investigate the cardiac effects and safety of paroxetine in healthy adults

**DOI:** 10.1002/bcp.70398

**Published:** 2026-01-14

**Authors:** Sven C. van Dijkman, Mathieu Félices, Bhaskar Pandurangavittal, Sanman Ghorpade, Caroline Easterbrook, Marcin Zabielski, Oscar Della Pasqua

**Affiliations:** ^1^ GSK London UK; ^2^ PhinC Development Massy France; ^3^ ICON plc Bangalore India; ^4^ GSK Mumbai India; ^5^ GSK Warsaw Poland; ^6^ Clinical Pharmacology & Therapeutics Group UCL London UK

**Keywords:** cardiac conductivity, concentration‐QT relationship, healthy volunteers, paroxetine, QTc interval prolongation

## Abstract

**Aims:**

Paroxetine is a selective serotonin reuptake inhibitor (SSRI), approved for treatment of major depressive disorder and anxiety disorders. Some SSRIs are known to prolong the QT interval; however, clinical evidence to establish a lack of association between paroxetine and corrected QT interval (QTc) prolongation is limited. Therefore, this study aimed to characterize the relationship between paroxetine concentration and QT/QTc interval following therapeutic doses in healthy individuals.

**Methods:**

This open‐label, single‐arm, dose‐escalating concentration‐QT study (NCT06065735) was performed in healthy adults (18–65 years) without a history of cardiac disease or pre‐diagnosed mood disorder. Eligible individuals (n = 38) received paroxetine 20 to 60 mg QD for 1 week per dose level. Paroxetine plasma concentrations and electrocardiogram recordings were monitored over a 12 h period on Days 1 (baseline), 7 (20 mg), 14 (40 mg) and 21 (60 mg).

**Results:**

Mean change from baseline in QTcF (ΔQTcF) fluctuated between −7.1 and +4.7 ms. However, diurnal variation was also observed without treatment. A linear regression model showed no clinically significant effect of paroxetine concentrations on ΔQTcF, with a weak slope of 0.0108 ms/ng/mL (90% CI: 0.01, 0.03) and maximum ΔQTcF of +0.42 ms (90% CI: −2.68, 3.52) at 60 mg QD, corresponding to a Cmax of 221.4 (95%CI: 179.6–272.8) ng/mL. Similarly, paroxetine did not affect the mean change in PR or QRS interval, or heart rate relative to baseline.

**Conclusions:**

Paroxetine does not prolong QTc interval in healthy individuals to any clinically meaningful extent at therapeutically relevant doses. This study supports the favourable cardiac safety profile of paroxetine.

**What is already known about this subject**

- Citalopram and escitalopram, which are selective serotonin reuptake inhibitors (SSRI) have been shown to prolong the QT interval.
- Since the early 1990s, the SSRI paroxetine has been approved for the treatment of major depressive disorder (MDD) and anxiety disorders; however, clinical evidence for the lack of association between paroxetine and corrected QT interval prolongation (QTc) prolongation has not been evaluated in a controlled setting.
- As an alternative to thorough QT (TQT) studies, concentration‐QT (C‐QT) studies provide insight into the magnitude of potential QT‐prolonging effects at therapeutically relevant exposures of the drug of interest.

**What this study adds**

- This C‐QT study showed that there is no association between therapeutically relevant concentrations of paroxetine (up to 60 mg QD) and QTc interval prolongation in healthy individuals.
- These results corroborate previously reported findings describing the absence of any QTc‐prolonging effect of paroxetine in patients.
- Paroxetine's favourable cardiac safety profile offers prescribers clarity about treatment choices for the clinical management of patients with MDD and anxiety disorders.

## 1 INTRODUCTION

Paroxetine is the most potent serotonin reuptake inhibitor (SSRI) clinically available, and is indicated for the treatment of major depressive disorder (MDD), anxiety disorders and, as a controlled release formulation, for pre‐menstrual dysphoric disorder.^1^, ^2^ Several SSRIs have been shown to have a low risk for QT interval prolongation, including sertraline, fluoxetine and fluvoxamine, with paroxetine showing the lowest risk.^3^, ^4^, ^5^ However, a review by Funk et al. found that there are variations in the definition of QT interval prolongation used in these studies, making it difficult to interpret findings.^6^ Among the SSRIs, only citalopram and escitalopram have been shown to have a clear QT‐prolonging effect,^3^, ^6^, ^7^, ^8^ which suggests that QT interval prolongation is not a SSRI class effect.^3^, ^8^, ^9^

Pre‐clinical studies have shown conflicting evidence regarding the potential effect of paroxetine on QT interval.^10^, ^11^, ^12^ An in vitro study by Lee et al. showed that paroxetine can block Ether‐à‐go‐go‐Related Gene (hERG) channels at clinically relevant plasma concentrations,^10^ while an in vitro and in silico study by Plijter et al. suggests paroxetine may act as an inhibitor of voltage‐gated sodium channels (Na_v_1.5) by influencing different electrophysiological characteristics of this ion channel.^12^ Furthermore, the authors concluded that the effects of paroxetine on the Na_v_1.5 ion channel may worsen the effects of loss‐of‐function mutations in the *SCN5A* gene and should be avoided for patients with long QT syndrome or Brugada syndrome.^12^ On the other hand, clinical studies have found that paroxetine therapy was not a significant risk factor for increased corrected QT interval (QTc)^6^, ^13^, ^14^, ^15^, ^16^, ^17^, ^18^ and no electrocardiogram (ECG) abnormalities were identified.^14^ Other studies performed in healthy individuals also showed no clear QTc interval prolongation signal with paroxetine use^3^, ^6^. Similarly, Okayasu et al. found no significant changes in QTc interval in patients with mood disorders who received a 22.5 mg mean dose of paroxetine (n = 129) between 2007–2010.^5^

At the time of first approval of paroxetine for the treatment of MDD in the early 1990s, there was no suspicion or data suggesting that it could affect heart conductivity and consequently, prolong QTc interval. In addition, there was no regulatory requirement to evaluate the potential effects on QTc interval. Hence, no dedicated clinical study was performed to examine this potential concern. However, a more recent review of literature and safety databases (data on file) found sparse cases reporting on QT prolongation occurring in the context of paroxetine use.^19^, ^20^, ^21^ These cases, in addition to meta‐analysis findings of QT prolonging effects by other SSRIs resulted in a suspicion of a potential association of QT prolongation with paroxetine, although a causal relationship could not be concluded.^7^ While this suspicion was relatively low in view of historical safety data and existing literature, it provided the rationale for conducting a controlled study into the potential effect of paroxetine on QT/QTc interval. In line with the International Council for Harmonization (ICH) of Technical Requirements for Pharmaceuticals for Human Use E14/S7B (2022) guidelines, a concentration‐QT (C‐QT) analysis can be considered in settings where the suspicion for a potential QT‐prolonging effect is deemed to be low,^22^, ^23^ and suggest that a study specifically designed to support a phase I concentration‐response analysis can sufficiently measure the effect of a drug on QTc interval, negating the need for a placebo comparator.^22^, ^23^, ^24^ Indeed, when a full ECG profile is performed before dosing commences, the participant's pre‐dosing data may function as its own control.

Given the importance to eliminate potential confounding factors (e.g., co‐morbidities, co‐medications) and establish whether previous reports suggesting the absence of clinically significant risk of QTc interval prolongation are supported by the underlying pharmacokinetic‐pharmacodynamic (PKPD) relationship, this C‐QT/QTc study aimed to characterize in a controlled manner the effect of systemic exposure to paroxetine following therapeutic dose levels and changes in QTc prolongation in healthy individuals.

## 2 MATERIALS AND METHODS

### 2.1 Study design

**FIGURE 1:**
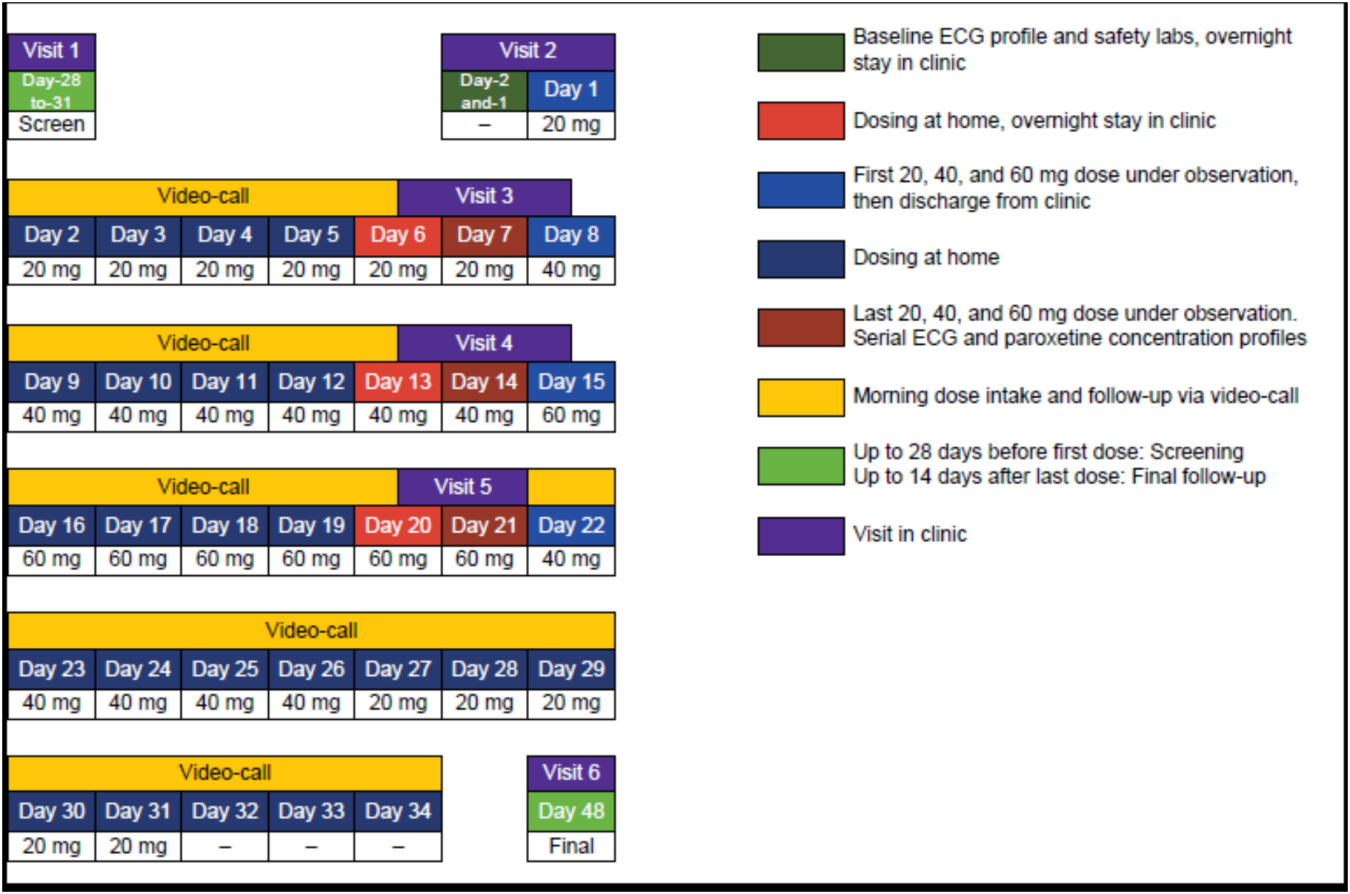
Study design. Each treatment period on a dose level (i.e., 20, 40 or 60 mg) has a duration of one week, with ECG monitoring and blood sampling for pharmacokinetics performed on the last (7th) day of each period. ECG monitoring is also performed over 12 h on day −1 prior to administration of the first 20 mg dose of paroxetine. Tapering of the dose (down‐titration) over a period of 10 days at the end of the dose escalation was used to prevent withdrawal symptoms.

This was an open‐label, single‐arm, dose‐escalating C‐QT study (NCT06065735) to investigate the ECG effects following increasing doses of paroxetine, as well as the safety and tolerability profile of paroxetine in healthy adults. Eligible individuals received paroxetine titrated at a dose of 20 mg QD, then 40 mg QD and then 60 mg QD over the course of a week at each dose level. Considering the elimination half‐life (of up to 24 h) of paroxetine, a week treatment at each dose level was assumed to be sufficient to ensure exposure at steady‐state. The study design is shown in Figure 1 and is further described in the Supporting Information. Vital signs and ECG were collected at on‐site visits at different time points on Days −1 (baseline), 7, 14 and 21 (Figure 1). During the treatment period, all laboratory samples and vitals (including one PK sample) were obtained prior to administration of the paroxetine dose.

The study was conducted in accordance with international guidelines including the Declaration of Helsinki, Council for International Organizations of Medical Sciences International Ethical Guidelines, applicable ICH good clinical practice guidelines, US 21 Code of Federal Regulations 312.120, and other applicable laws and regulations. Individuals were informed that their participation was voluntary, and all individuals signed a statement of informed consent that met the requirements of 21 Code of Federal Regulation 50, local regulations, ICH guidelines and the independent ethics committee or study site.

### 2.2 Study population

Healthy adult volunteers (aged 18–65 years), who were non‐smokers with no history of cardiac abnormalities or mood disorders, were included. Females were eligible to participate if they were of non‐childbearing potential. All individuals who received at least one dose of the study intervention were included in the safety analysis set. All individuals in the safety analysis set who had at least one concentration measurement were included in the pharmacokinetic analysis set. All individuals in the safety analysis set who had at least one ECG assessment were included in the pharmacodynamic analysis set. The full list of eligibility criteria can be found in the Supporting Information.

### 2.3 Study objectives

The primary objective of this study was to evaluate the potential effect of oral doses of 20, 40 and 60 mg paroxetine on QTc with Fridericia's correction (QTcF), as measured by the change from baseline in QTcF (ΔQTcF). The secondary objective was to assess the safety and tolerability of paroxetine. Secondary endpoints included the occurrence of adverse events (AEs), serious AEs (SAEs), haematological and clinical laboratory tests and vital signs.

### 2.4 Sample size

A linear mixed‐effects model was used to determine the sample size.^25^ A Monte Carlo simulation analysis was used to determine the probability of study success depending on various levels of assumed concentration‐QTc correlation; 0 ms, 5 ms and 10 ms of true effect at the Cmax of the 60 mg QD dose. For each scenario, it was determined whether the estimated 90% upper limit of the confidence interval (CI) of ΔQTc at the geometric mean of the 60 mg dose encompassed the 10 ms cut‐off value. Based on this approach, it was expected that under the assumption of no effect of paroxetine on QTc interval at the 60 mg dose, the probability of incorrectly detecting an effect (90% upper limit of the CI ≥ 10 ms) was <1% (false positive) if 36 individuals were enrolled or <5% in case of a 5 ms true effect. Conversely, the power to appropriately detect a true 10 ms effect based on 36 individuals was found to be >80%.

### 2.5 ECG recordings

Standard 12‐lead ECGs were recorded using the same make of device (Mortara Surveyor Telemetry Central and Mortara telemetry transmitters Model: Surveyor S4), except for the screening visit, where CardioSoft was used. All ECG traces were reviewed by a licensed cardiologist. ECGs were required to be recorded after a 10‐minute resting period in the supine position to ensure a stable baseline. All ECGs were performed in triplicate collected within 5 min, with at least 1 min in between each timepoint, and the mean value of the QTc interval calculated for each individual. A single ECG trace (in triplicate) was collected at: screening and at withdrawal/discontinuation and follow‐up/end of study (up to 14 days after last treatment dose). Serial (triplicate) ECG traces were collected on Day −1, Day 7, Day 14 and Day 21 at the following timepoints: 15 min pre‐dose (−0.25 h) and post‐dose; and at the following timepoints: 1, 2, 3, 4, 4.5, 5, 5.5, 6, 8, 10 and 12 h. ECG recordings were paired with blood sampling for paroxetine concentrations but obtained before the actual sample collection to avoid changes in autonomic tone associated with the psychological aspects of blood collection.

### 2.6 Pharmacokinetic analysis

Blood samples (3 mL each) were collected for the measurement of paroxetine plasma concentrations at baseline (Day −1 at −0.25 h [pre‐dose]), and on Day 7, Day 14 and Day 21 at the following timepoints: −0.25, 1, 2, 3, 4, 4.5, 5, 5.5, 6, 8, 10 and 12 h. Blood samples were centrifuged, and plasma separated and stored at −80 °C. Samples were analysed using liquid chromatography tandem mass spectrometry validated to be linear across a concentration range of 0.5 – 500 ng/mL. The maximum observed concentration (C_max_) and time of C_max_ (T_max_) at steady‐state were derived as metrics of exposure using non‐compartmental analysis in Phoenix WinNonlin (WNL) version 8.3 (Certara, Radnor, Pennsylvania, United States).

### 2.7 Statistical methods

A linear mixed‐effects model was used to analyse any potential relationship between paroxetine concentrations and QTcF interval, as described previously.^25^ The model was fitted to paroxetine concentrations using change in QTc interval from baseline (ΔQTc) data pairs, as follows:

(Eq 1) $${\text{ΔQTc}}_{i,k}=\left(\theta_{0}+\eta_{0,i}\right)+\left(\theta_{1}+\eta_{1,i}\right)*C_{i,k}+\theta_{2,k}*{\text{TIME}}_{i,k}+\theta_{3}\left({\mathrm{QTc}}_{i,k=0}-\bar{{\mathrm{QTc}}_{0}}\right)+\varepsilon_{i,k}$$

with i denoting the subject, k the nominal time, θ_0_ the population mean intercept at concentrations of 0, θ_1_ the population average slope of the linear association between concentration and ΔQTc, C_i,k_ the concentration for subject i at time k, θ_2,k_ the fixed effects associated with each nominal time (as categorical variables), θ_3_ the fixed effect associated with the centred baseline, QTc_i,k = 0_ – mean (QTc_i,k__= 0_) the baseline centred by the mean over all individuals i, and η the inter‐individual variabilities (random effects) associated with intercept (θ_0_) and slope (θ_1_), respectively. ε_i,k_ is the residual error for subject i at time k.

Assumptions underlying the model were checked, such as the drug effect on heart rate, appropriateness of the QTc correction factor, the nature of the direct effect and the evidence for a linear relationship. In this model, ΔQTc was the dependent variable; paroxetine concentration and time after dose and baseline were independent variables. Between‐individual random effects with unstructured covariance matrix were associated with the intercept (θ_0_) and the slope (θ_1_) parameters. The parameters for correction at each timepoint (θ_2_) were used to account for any variation in QTc across the day, these parameters varied for each matched timepoint but were kept the same across treatment periods. Parameter θ_3_ is the fixed effect associated with the difference of each baseline value relative to the overall mean. The pre‐dose (i.e., at time −0.25 h) QTcF value on each serial ECG day (Day −1, Day 7, Day 14 and Day 21) served as the reference (control) to calculate ΔQTcF across the ECG profile for each of those days.

ΔQTc (with 90% CI) was predicted from the model at the geometric mean C_max_ observed at the highest dose (60 mg). If the upper bound of 90% CI for this predicted change in QTc interval fell below 10 ms, this indicated that there was no clinically relevant QTc prolongation up to and including paroxetine doses of 60 mg. If the upper 90% CI limit ≥ 10 ms, the same evaluation was to be performed for the ΔQTc at the geometric mean C_max_ of the 40 mg QD dose. Diagnostic and goodness‐of‐fit plots were generated for the final model, including quantile‐quantile plots of residuals; concentrations *vs*. residuals; time and baseline *vs*. residuals; model‐predicted ΔQTc *vs*. observed ΔQTc plotted with a Loess line; and mean observed and predicted ΔQTc by deciles of concentrations (quantile plot) with model slope and 90% CI.

Continuous data were summarized descriptively, unless otherwise stated. Categorical data were summarized in terms of the number of individuals providing data at the relevant time point (n), frequency counts and percentages. All report outputs were produced using SAS version 9.4 (Cary, North Carolina, USA). A copy of the model code in SAS is provided in the Supporting Information.

### 2.8 Nomenclature of targets and ligands

Key protein targets and ligands in this article are hyperlinked to corresponding entries in http://www.guidetopharmacology.org, and are permanently archived in the Concise Guide to PHARMACOLOGY 2023/24.^26^

## 3 RESULTS

### 3.1 Study population

An overview of the baseline characteristics of the study population and treatment allocation is presented in Table 1. A total of 122 healthy individuals were screened, of which 38 were enrolled in this study. All 38 individuals received the 20 mg dose of paroxetine, 33 (87%) received the 20 and 40 mg doses, and 32 (84%) received the 20, 40 and 60 mg doses. The mean age (standard deviation) of individuals was 37.3 (11.5) years, 61% (n = 23) were male and 66% (n = 25) were White. The most frequently reported concomitant medication was paracetamol, and no concomitant medications received by study individuals were considered to affect the results of the study. Seven (18%) individuals discontinued the study intervention; five individuals discontinued at 20 mg, one at 40 mg and one at 60 mg. Two participants had paroxetine concentration values below the lower limit of quantification for all blood samples (i.e., one participant at the 40 and 60 mg doses, and another participant at the 60 mg dose), even though these participants took paroxetine doses as planned in the protocol. Paroxetine concentrations for these timepoints were set to zero, but to avoid introducing bias, these data were not included in the C‐QT analysis.

**TABLE 1:** Baseline characteristics and study disposition.

Characteristic	Overall (N = 38)
Age, years, mean (SD)	37.3 (11.5)
Male, n (%)	23 (61)
Race, n (%)	
White	25 (66)
Asian	7 (18)
Black or African American	5 (13)
Mixed race	1 (3)
Ethnicity, n (%)	
Not Hispanic or Latino	35 (92)
Hispanic or Latino	3 (8)
Body mass index (kg/m^2^), mean (SD)	23.8 (2.8)
Medical history by SOC, n (%)	
Immune system disorders	12 (32)
Infections and infestations	6 (16)
Injury, poisoning and procedural complications	5 (13)
Respiratory, thoracic and mediastinal disorders	5 (13)
Surgical and medical procedures	5 (13)
Prior and concomitant therapy, n (%)^a^	
COVID‐19 vaccine	13 (34)
Paracetamol	6 (16)
Contraceptive products^b^	4 (11)
Study disposition, n (%)	
Enrolled in the study	38 (100)
Paroxetine 20 mg	38 (100)
Paroxetine 40 mg	33 (87)
Paroxetine 60 mg	32 (84)
Discontinued^c^	7 (18)

^a^ Only comedications used by at least 10% of study individuals. ^†^Oral (n = 2), subdermal (n = 1) and intrauterine (n = 1) contraceptives. ^‡^Four individuals were discontinued from the study due to the occurrence of an SAE (dyskinesia [one individual]) or AEs (including COVID‐19 [three individuals]); one individual was discontinued due to use of cocaine, one individual was withdrawn due to non‐availability and one due to withdrawal by individual and AE (confusional state). AE, adverse event; SD, standard deviation; SAE, serious AE; SOC, system organ class.

### 3.2 Observed paroxetine concentrations, change in QTcF interval and heart rate from baseline

**FIGURE 2:**
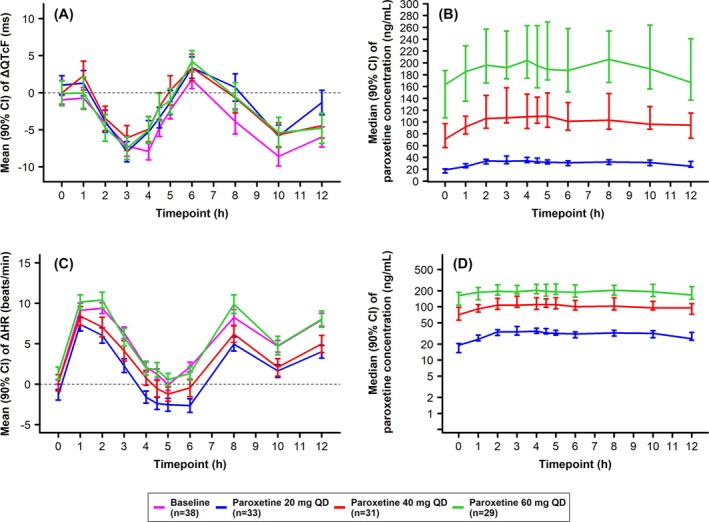
Changes in QTcF interval and heart rate from baseline stratified by paroxetine dose. (A) Mean change from baseline (95% CI) in QTcF interval. (B) Median (95% CI) paroxetine concentrations at steady‐state over time after dose. (C) Mean change from baseline (95% CI) in heart rate (beats/minute) over time (D) median (95% CI) paroxetine plasma concentration at steady state over time after dose on a semi‐logarithmic scale. Data presented based on the C‐QT analysis set, which excluded observations for one individual at the 40 mg QD paroxetine dose and two individuals at the 60 mg QD paroxetine dose due to BLQ paroxetine concentrations at the respective doses. Δ, change; CI, confidence interval; h, hours; QTcF, QT interval with Fridericia's correction.

Initially, graphical summaries and descriptive statistics of the observed paroxetine concentrations were generated along with the change in QTcF interval and heart rate from baseline (i.e., ΔQTcF and ΔHR). As shown in Figure 2, ΔQTcF oscillated irrespective of dose level or paroxetine concentrations in plasma. The same trend was also observed during the ECG measurements at baseline (Day −1), suggesting that they were due to diurnal fluctuations in QT interval as noted elsewhere in the literature.^27^ The mean ΔQTcF oscillated between −7.1 ms (90% CI: −9.58, −4.58) and 4.7 ms (90% CI: 2.06, 7.25) (Figure 2a). On Days 7 (20 mg), 14 (40 mg) and 21 (60 mg), the mean change in heart rate fluctuated similarly during the 12‐h recording period, according to a diurnal variation pattern (Figure 2c) and no relation to dose could be discerned. Administration of paroxetine did not appear to affect mean ΔQTcF (Figure 2a) but did slightly increase heart rate; however, this was not to a clinically relevant degree (Figure 2c). Median plasma paroxetine concentrations are summarized by nominal time point on linear (Figure 2b) and semi‐logarithmic scales (Figure 2d). Median peak concentrations (Cmax) following doses of up to 60 mg paroxetine occurred between 2 and 4 h post‐administration. The geometric mean Cmax for doses 20 mg (Day 7), 40 mg (Day 14) and 60 mg (Day 21), were 36.2, 128.2 and 221.4 ng/mL, respectively. The median Tmax was 4.04, 3.08 and 3.05 h, respectively (Figure 2b and Figure 2d).

**FIGURE 3:**
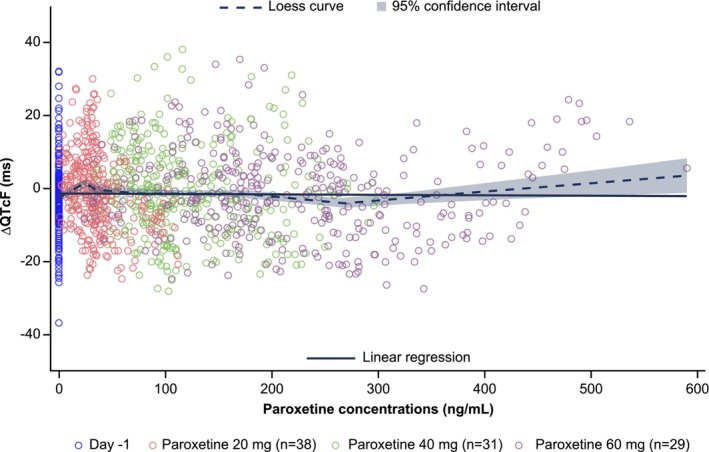
Scatter plot describing the change in QTcF interval from baseline across increasing paroxetine steady‐state concentrations in plasma. Dashed line depicts a trend curve (Loess). Grey area: 95% confidence interval. Each circle is an individual paroxetine plasma concentration. Open blue circles describe the variation in ΔQTcF over a 12 h period prior to treatment (day −1). Δ, change; QTcF, QT interval with Fridericia's correction.

For the sake of thoroughness, ΔQTcF was plotted against all paroxetine concentrations in a pairwise manner, including the data collected at baseline on Day −1 (i.e., no treatment). As shown in Figure 3, no apparent correlation is observed. Inspection of the Loess curve does not suggest any significant correlation or trend either. The concentrations observed above 480 ng/mL are from a single participant and are insufficient to support a nonlinear component to any potential C‐QT correlation. It is likely an artefact of physiological variation in ΔQTcF as observed across the baseline and overall concentration range, and indicated by the trend line.

### 3.3 Linear mixed effects modelling of paroxetine concentrations *vs.* change in QTcF interval from baseline

**FIGURE 4:**
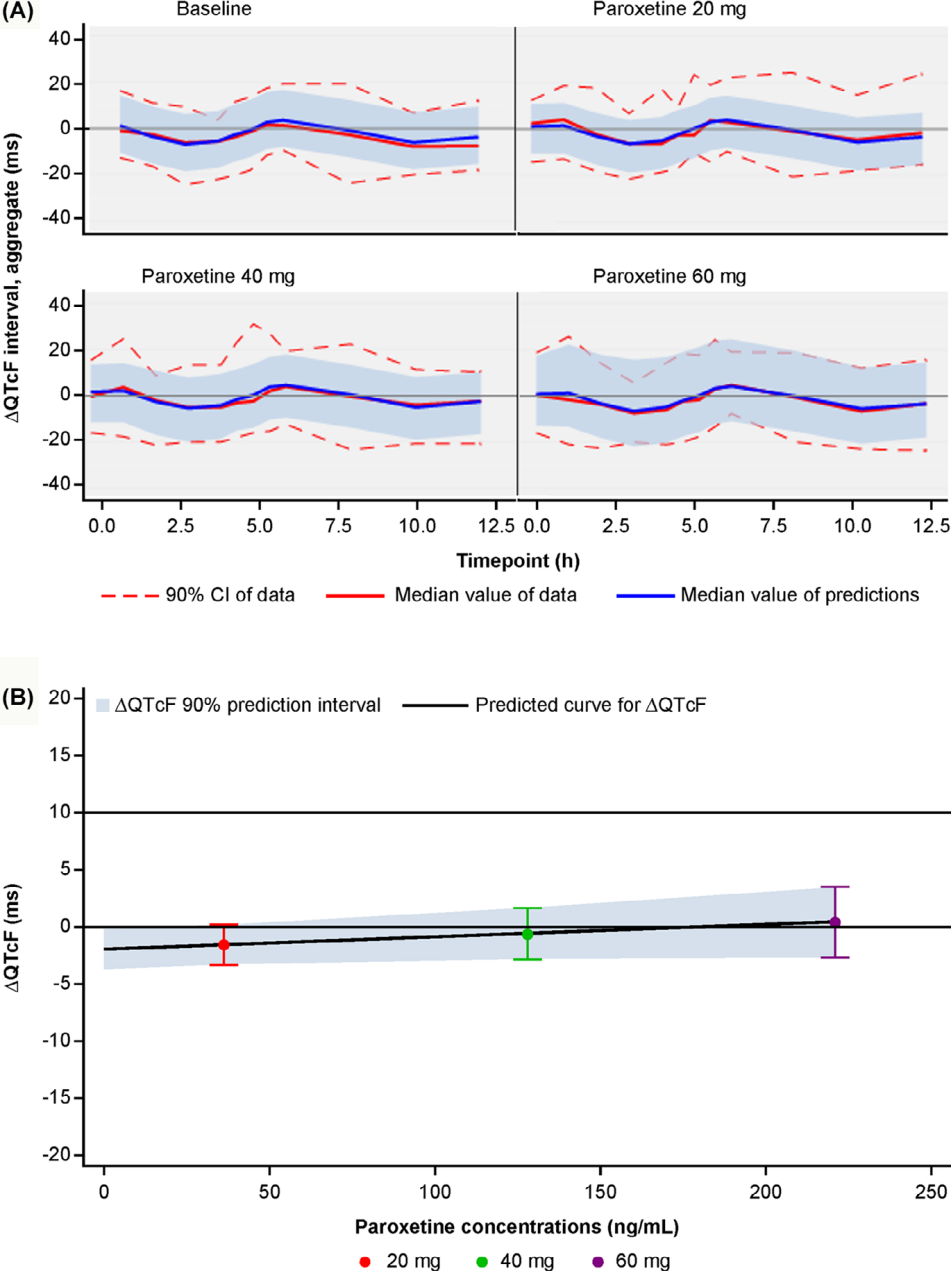
Change in QTcF from baseline. (A) Goodness‐of‐fit plots, blue and red lines depict the median predicted and observed ΔQTcF, respectively. Shaded area and dashed red lines represent the 90% prediction interval, and 5th and 95th percentiles of the observed data, respectively. (B) Regression line describes the model‐predicted ΔQTcF at geometric mean C_max_ for each dose. Shaded area represents the 90% prediction interval. Δ, change; CI, confidence interval; h, hours; QTcF, QT interval with Fridericia's correction.

The linear mixed‐effects model adequately fit the paroxetine concentrations *vs*. ΔQTcF data, as shown by the goodness‐of‐fit and other relevant diagnostic plots (Figure 4, Figures S1 to S7). ΔQTcF showed a diurnal change over time, which was present both before and during paroxetine exposure, as shown in Figure 4a when comparing the baseline panel *vs*. paroxetine at 20, 40 and 60 mg QD panels. As historically, this diurnal variation is accounted for using fixed effects for each timepoint, the model implementing such an approach is reported here. The slope describing the relationship between paroxetine concentration and ΔQTcF was weak (slope [95% CI]: 0.0108 [0.00, 0.03] ms/ng/mL) and not statistically different from 0, thus confirming the absence of a relationship between paroxetine concentrations resulting from doses up to 60 mg and ΔQTcF (Table 2). Using the final model estimates, the mean predicted ΔQTcF for the mean C_max_ at the paroxetine 60 mg dose was 0.42 ms (standard error: 1.8 ms) with an upper 90% prediction interval of 3.5 ms, which remained well below the limit of 10 ms, showing no statistically significant or clinically meaningful QTcF prolongation (Figure 4b). In addition, an attempt was made to assess the magnitude of random time variation at each sampling time. Parameter estimates for mean predicted ΔQTcF at each time (from 1 h to 12 h post dose) corroborate the findings obtained at Cmax. (Table S1), i.e., that ΔQTcF is not significantly affected by increasing paroxetine concentrations. The 90% confidence intervals included 0 at all time points, except for 5.5 and 6.0 h after dose, when a significant decrease in heart rate relative to baseline was also observed (Figure 2c).

**TABLE 2:** Model parameter estimates and predicted change in QTcF interval from baseline at Cmax* at steady‐state following different dose levels of paroxetine.

	Estimate (SE)	95% CI
Fixed effects parameters		
Intercept (off‐drug) (ms)	0.66 (1.23)	−1.78, 3.10
Baseline QTcF (ms)	−0.15 (0.06)	−0.28, −0.02
Paroxetine slope (ms/ng/mL)	0.01 (0.01)	0.00, 0.03
Predicted change in QTcF (ms)		
40 mg (C_max_ GM: 128.2 ng/mL) (n/N, 31/38)	−0.58 (1.34)	−2.85, 1.68
60 mg (C_max_ GM: 221.4 ng/mL) (n/N, 29/38)	0.42 (1.82)	−2.68, 3.52

Observations for one individual at 40 mg and two individuals at 60 mg were excluded from the analysis. Equation 1: ΔQTc_𝑖,𝑘_=(θ_0_ + η_0,𝑖_) + (θ_1_ + η_1,𝑖_)*𝐶_𝑖,𝑘_+θ_2_,_k_*𝑇𝐼𝑀𝐸_𝑖,𝑘_+θ_3_(𝑄𝑇𝑐_𝑖,𝑘__=0_−𝑄𝑇𝑐_0_)+ 𝜀_𝑖,𝑘_).

C_max_, maximum concentration; GM, geometric mean; QTcF, QT interval with Fridericia's correction; SE, standard error.

* Geometric means and 95%‐confidence intervals for Cmax were:

**Table:** 

Dose level	Geometric mean (ng/mL)	95% CI (ng/mL)
20 mg	36.2	29.4–44.6
40 mg	128.2	108.3–151.8
60 mg	221.4	179.6–272.8

### 3.4 Safety profile and clinical laboratory tests

Table 3 shows a summary of the reported adverse events (AEs). Overall, 29 individuals (76%) reported 139 AEs; all AEs were either mild (n = 120) or moderate (n = 19) in severity. A total of 27 individuals (71%) reported 120 AEs that were related to the study intervention. Four individuals were discontinued from the study, either due to the occurrence of a serious adverse event (SAE) (one; dyskinesia) or AEs (two individuals tested positive for COVID‐19 and one individual with coronavirus disease). One individual (3%) who received paroxetine 20 mg had an SAE of dyskinesia, which was considered possibly related to study medication and led to discontinuation of the study intervention and withdrawal of the individual from the study. After discontinuation of paroxetine, the dyskinesia resolved. The most common AEs were headache (32%, n = 12), fatigue (24%, n = 9) and nausea (24%, n = 9). There were no deaths or pregnancies reported in this study.

**TABLE 3:** Summary of all‐cause adverse events.

AE category	Paroxetine	Paroxetine	Paroxetine	Overall	Tapering phase/
(n, %)	20 mg (n = 38)	40 mg (n = 33)	60 mg (n = 32)	(N = 38)	follow‐up (N = 38)
Number of AEs	23 (61)	19 (58)	9 (28)	29 (76)	13 (34)
Most common AEs by preferred term					
Headache	6 (16)	5 (15)	2 (6)	12 (32)	5 (13)
Fatigue	5 (13)	3 (9)	1 (3)	9 (24)	1 (3)
Nausea	6 (16)	3 (9)	0 (0)	9 (24)	4 (11)
Somnolence	4 (11)	4 (12)	1 (3)	6 (16)	0
Insomnia	4 (11)	1 (3)	2 (6)	6 (16)	2 (5)
Dizziness	4 (11)	1 (3)	0 (0)	5 (13)	6 (16)
Decreased appetite	4 (11)	1 (3)	0 (0)	5 (13)	1 (3)
Sexual dysfunction	0	2 (6)	3 (9)	5 (13)	0
Any SAE	1 (3)	0	0	1 (3)	0
Drug‐related AE	22 (58)	18 (55)	8 (25)	27 (71)	11 (29)
AE leading to discontinuation^a^	2 (5)	1 (3)	1 (3)	4 (11)	0
Death	0	0	0	0	0

^a^ Four individuals were discontinued from the study due to the occurrence of an SAE (dyskinesia) or AEs (including COVID‐19 [two individuals] and coronavirus disease); one individual was discontinued due to use of cocaine, one individual was withdrawn due to non‐availability, and one due to withdrawal by individual and AE confusional state.

AE, adverse event; SAE, serious adverse event.

There were no clinically significant abnormalities or variation in ECG parameters other than some fluctuation in QTcF. Two individuals (one for the 20 mg dose and one for the 40 mg dose) had a QTcF >450 msec but ≤480 msec, whereas there were four individuals (three for the 40 mg dose and one for the 60 mg dose) with an increase from baseline exceeding 30 msec but ≤60 msec (Table S2). These effects were not dose or drug related and appear to be incidental. In this study, a few treatment‐emergent morphological abnormalities were observed. However, these were considered incidental, unrelated to paroxetine and not clinically significant (Table S3). There were no clinically meaningful findings in the haematology, clinical chemistry, coagulation and vital signs measurements, or other observations related to the safety profile of paroxetine.

## 4 DISCUSSION

Whilst prescribing information for newer medicines is supported by the evaluation of the pro‐arrhythmic risk and eventual details on the underlying concentration‐QT effect relationship prior to market authorization,^28^ such data were not generated for paroxetine at the time of its approval. In contrast to controlled clinical studies, pharmacovigilance data on drug use in the target patient population often reflect associations, providing incomplete or limited insight into the relationships between drug exposure and effect. Here we have attempted to generate data supporting causal inference, i.e., to establish whether increasing concentrations of paroxetine within the therapeutically recommended dose range are associated with QTc interval prolongation.^28^, ^29^ We do not delve into further discussions about whether QT_c_ interval prolongation, baseline QT interval values, or pre‐existing cardiac conditions are the best predictors of arrhythmic events and cardiovascular risk, and as such may partly explain some of the case reports associated with the use of paroxetine.^29^, ^30^ Consequently, in this study we employed a C‐QTc analysis to establish that exposure to therapeutic doses of paroxetine does not have a clinically relevant effect on QT interval, as assessed by changes in QTcF interval relative to baseline (ΔQTcF). Our evaluation included the highest recommended dose of 60 mg QD, which corresponds to steady‐state maximum concentrations of 221.4 (95%CI: 179.6–272.8) ng/mL. This range is over two‐fold the concentrations observed after administration of the typically recommended doses of 20 mg or 40 mg QD, depending on the indication.^31^ It should also be noted that the pharmacokinetics of paroxetine observed in our study was consistent with previous reports.^32^ Even though study participants were not genotyped for CYP2D6 polymorphism, the observed interindividual variability in plasma concentrations seems to reflect the contribution of other concurrent factors, such as age, sex and body weight, which have been identified as covariate factors on the clearance of paroxetine.^33^

In fact, the current results corroborate previous investigations on paroxetine, which indicate no evidence of QTc interval prolongation,^34^ including a meta‐analysis of patient data, where 95% confidence intervals for the difference between the effect of paroxetine *vs*. placebo on QTc interval ranged from −5.76 to + 3.68 ms.^7^ It also reflects the current classification of paroxetine by Arizona CERT (AZCERT®), an non‐profit organization focused on the safe use of medicines, which provides information on the association of different drugs with varying risks of torsade de pointes (TdP). Paroxetine shows a weak association with TdP, unlikely at therapeutic doses.^35^ Nevertheless, it should be clear to readers that a patient's risk of QTc prolongation depends not only on medications taken, but also on individual (intrinsic) and other environmental (extrinsic) factors.^30^, ^34^, ^36^, ^37^ Our study provides conclusive insight into the effect of systemic exposure to paroxetine at therapeutically relevant doses. Given these findings, it is possible to distinguish the effect of drug‐induced QT interval prolongation from other concurrent factors, such as age over 65 years, female sex (longer QTc interval than men and twice the risk of drug‐induced TdP); myocardial hypertrophy (e.g., in arterial hypertension); congenital QT syndrome; bradycardia (leads per se to QTc prolongation; sinus bradycardia; 2nd‐ and 3rd‐degree atrioventricular block); electrolyte disturbances (hypokalaemia, hypomagnesaemia); and eventual overdose, intoxication or metabolic inhibition by concomitantly administered drugs and/or reduced drug clearance.^34^, ^38^, ^39^, ^40^

In light of the current findings, it is of interest to understand why some pre‐clinical protocols have indicated that paroxetine may act as an inhibitor of cardiac Na_V_1.5 channels and potentially affect QTc interval.^10^, ^11^, ^12^ Very often such discrepancies arise from the lack of a quantitative translational framework to assess the implications of affinity, binding and potency from in vitro settings to humans.^36^ For instance, dofetilide displacement experiments with cisapride, moxifloxacin and sotalol revealed that binding curves are unrelated to the in vivo potency estimates for QTc interval prolongation in dogs and humans.^41^ Likewise, lamotrigine was found to block peak and late Na_V_ 1.5 current at therapeutically relevant exposure, with rapid kinetics and biophysical properties similar to mexiletine. However, no clinically meaningful prolongation in QT, QRS or PR interval was observed in healthy subjects, supporting the clinical safety of lamotrigine in patients suffering from epilepsy and bipolar disorder.^42^ On the other hand, there are cases, where interactions may occur at pharmacodynamic level, as with tamoxifen, a drug known to prolong QT interval. Tamoxifen is not only a CYP2D6 substrate but also an inhibitor of repolarizing potassium currents (I_kr_), which may explain the pronounced effect on QTc interval when administered in combination with SSRIs, including paroxetine.^40^ Unfortunately, the authors did not measure blood concentrations to assess the magnitude of the pharmacokinetic interaction. Similarly, previous publications in which paroxetine use has been associated with QTc interval prolongation in patients, it remained unclear whether paroxetine contributed directly or indirectly to the observed  increases in QTc interval due to a potential pharmacokinetic (or pharmacodynamic) interaction.^6^, ^32^, ^39^

In summary, our results support the view that in the absence of a pro‐arrhythmic effect or any obvious physiological effect on cardiac conductivity, it is unlikely that paroxetine contributes to signals in patients who are more prone to experience QTc interval prolongation, such as reported in patients with heart failure with preserved ejection fraction.^43^ In addition, from a clinical safety perspective, there were no new safety signals. The AEs reported in this study were consistent with the known safety profile of paroxetine.^31^ There were no clinically meaningful findings in the haematology, clinical chemistry, coagulation and vital signs measurements or other observations related to safety in this study. Paroxetine showed no clinically significant effect on any other ECG parameters, which aligns with the results reported in a previous clinical study,^14^ and a few treatment‐emergent morphological abnormalities were observed. Paroxetine had no clinically meaningful effect on heart rate in healthy volunteers, contradicting a previous study, which showed that paroxetine can reduce heart rate; however, that study only involved four healthy male individuals.^44^ It should be noted that the higher frequency of AEs at the lower doses reflects the development of tolerance to SSRI effects after repeated dosing, even though a few dropouts did occur across higher doses due to tolerability.

Regardless of the strength of the study design, which aimed to generate data in support of a C‐QT analysis in lieu of a less efficient, standard thorough QTc (TQT) study^24^, ^25^, we acknowledge a few limitations. First, we have carefully considered the ethical implications of exploring supratherapeutic doses and decided that such effects could be better evaluated through the use of extrapolation, taking into account the estimated slope of the concentration‐QT interval relationship across the therapeutically recommended doses. We also understand that the inclusion of positive control (e.g., moxifloxacin) might have provided direct evidence of the sensitivity of participants to delayed ventricular repolarization.^45^ However, mutations affecting ventricular repolarization are rather rare and as such unlikely to affect the overall results or conclusions from the current study. While no separate placebo arm was included in this study, serial ECG measurements were collected over 12 h prior to the start of treatment (Day −1). This enabled within‐subject characterization of diurnal fluctuation in QTcF interval and heart rate. As noted by Täubel and colleagues, the ability to observe meal effect or diurnal changes in ΔQTcF may serve as evidence of assay sensitivity.^46^ Consequently, we do not expect any bias or inaccuracies in the estimation of drug‐specific parameters based on the data obtained at steady state on treatment days 7, 14 and 21. Furthermore, any period effect would have been picked up as a bias in model predictions *vs*. observed data during model development. Lastly, it should be highlighted that the choice for healthy volunteers rather than patients may be seen as a limitation. Given the role of co‐morbidities and co‐medications with potential effect of ventricular repolarization, it is unclear whether some findings can be extrapolated to patients with a history of cardiovascular events or mood disorders who may have underlying pathologies that make them more sensitive to QTc‐prolonging effects. Yet, the absence of confounding allowed us to establish how increasing concentrations of paroxetine affect the QTc interval, without the problem of the high false‐positive rate in TQT studies employing traditional statistical analysis.^28^

## 5 CONCLUSIONS

No evidence was detected that doses of paroxetine up to 60 mg QD yield clinically relevant prolongation of the QT interval in healthy volunteers. In line with previous meta‐analyses of the effect of paroxetine on the QTc interval in patients, these results provide additional evidence for its cardiac safety profile. Despite in vitro data showing ion channel binding, such finding does not translate into a clinically relevant effect on the QTc interval and, hence, do not suggest drug‐induced alteration of cardiac conductivity. In addition, the safety findings in this study were consistent with the known safety profile of paroxetine. To determine whether these results apply to the same degree in all patients, future studies may investigate the risk of QTc prolongation with paroxetine among individuals with a history of cardiovascular events or mood disorders.

## AUTHOR CONTRIBUTIONS

ODP and SCvD designed the investigation protocol, MF and SCvD performed the analysis. SCvD, BP, SG, CE, MZ and ODP contributed to interpretation of the data, writing the manuscript and approved its submission for publication.

## CONFLICT OF INTEREST STATEMENT

SvD, SG, CE, MZ and ODP are employed by, and hold financial equities in, GSK. MF is an employee of PhinC Development, which received funding from GSK for this analysis. BP was contracted by GSK at the time of the study.

## Supporting information


**Table S1.** Predicted effect of random variation (i.e., time effect) at each sampling timea.
**Table S2.** Effect of paroxetine on ECG measures.
**Table S3.** Effect of paroxetine on morphological abnormalities.
**Figure S1.** Final model diagnostic plot. Predicted ΔQTcF *vs.* conditional studentized residuals.
**Figure S2.** Final model diagnostic plot. Paroxetine concentrations *vs.* conditional studentized residuals.
**Figure S3.** Final model diagnostic plot. Baseline ΔQTcF *vs.* conditional studentized residuals.
**Figure S4.** Final model diagnostic plot. Sampling time after dose *vs.* conditional studentized residuals.
**Figure S5.** Final model diagnostic plot. Treatment level *vs.* conditional studentized residuals.
**Figure S6.** Final model diagnostic plot. QQ plot of the distribution of conditional studentized residuals.
**Figure S7.** Final model diagnostic plot. Observed *vs*. conditional predicted ΔQTcF.

## Data Availability

The data that support the findings of this study can be requested through GSK's Clinical Study Register (ttps://www.gsk-studyregister.com). The data are not publicly available due to privacy or ethical restrictions.
