# Supplementary material for: An open‐label, single‐arm, dose‐escalating concentration–QT study to investigate the cardiac effects and safety of paroxetine in healthy adults
Source: Br J Clin Pharmacol. 2026 Jan 14;92(5):1385–96. doi: 10.1002/bcp.70398 (PMC13122290; doi:10.1002/bcp.70398)
Supplement: Supplementary file 1 — Table S1. Predicted effect of random variation (i.e., time effect) at each sampling timea. Table S2. Effect of paroxetine on ECG measures. Table S3. Effect of paroxetine on morphological abnormalities. Figure S1. Final model diagnostic plot. Predicted ΔQTcF vs. conditional studentized residuals. Figure S2. Final model diagnostic plot. Paroxetine concentrations vs. conditional studentized residuals. Figure S3. Final model diagnostic plot. Baseline ΔQTcF vs. conditional studentized residuals. Figure S4. Final model diagnostic plot. Sampling time after dose vs. conditional studentized residuals. Figure S5. Final model diagnostic plot. Treatment level vs. conditional studentized residuals. Figure S6. Final model diagnostic plot. QQ plot of the distribution of conditional studentized residuals. Figure S7. Final model diagnostic plot. Observed vs. conditional predicted ΔQTcF. [file BCP-92-1385-s001.pdf]

**Supporting Information**

**Study design**

The study design is shown in **Figure 1.** On Visit 1 (Days -28 to -31) all individuals attended a study site visit at screening. Visit 2 (Day -1) baseline (pre-dose) assessments were completed and the first dose of paroxetine (20 mg QD) was administered (Day 1). On Visit 3 (Day 7), serial electrocardiogram (ECG) and paroxetine concentration were measured. On Visit 3 (Day 8), the 40 mg QD dose was administered. On Visit 4 (Day 14), serial ECG and paroxetine concentration were measured. On Visit 4 (Day 15), the 60 mg QD dose was administered. On Visit 5 (Day 21), serial ECG and paroxetine concentration were measured. Visit 6 (up to 14 days after last 20 mg dose) was the Exit visit. Dose administration from Day 2–6, Day 9–13, and Day 16–20, occurred at home. Tapering from 60 mg to 40 mg QD occurred on Day 22–26, 40 mg to 20 mg QD on Day 27–31, 20 mg on Day 1–7 and Days 27–31 QD, and no dose final visit on Day 48. If individuals were not able to withdraw from 20 mg QD directly due to withdrawal effects, an additional tapering dose step of 10 mg QD was given for 5 days at the discretion of the investigator.

**Eligibility criteria**

*Inclusion criteria*

- Healthy male and female individuals between 18– 65 years old
- Female individuals were eligible to participate if they were of non-childbearing potential, defined as pre-menopausal females with a documented tubal ligation or hysterectomy; or postmenopausal defined as 12 months of spontaneous amenorrhea [in questionable cases a blood sample with simultaneous follicle stimulating hormone >40 IU/L and oestradiol <40 pg/mL (<147 pmol/L) was confirmatory]
- Individuals who had aspartate transferase, alanine transaminase, alkaline phosphatase, and bilirubin ≤1.5 × upper limit of normal (ULN; isolated bilirubin >1.5×ULN was acceptable if bilirubin was fractionated and direct bilirubin <35%)
- Individuals who had body weight ≥45 kg and body mass index within the range 18.0–29.5 kg/$m^2$
- Individuals who had no significant abnormality on 12-lead ECG at screening in supine position, including the specific requirements

- Individuals who could give written informed consent
- Individuals who were non-smokers (never smoked or not smoking for >6 months with <10 pack years history (Pack years = [cigarettes per day smoked/20]×number of years smoked or light smokers [less than five cigarettes per day])

*Exclusion criteria*

- Individuals with a history or presence of any medically significant disease or any disorder that would have introduced additional risk or interfered with the study procedures or outcome. In particular, a family history of QT prolongation, early or sudden cardiac death, or early cardiovascular disease
- Individuals with a history of symptomatic arrhythmias, hypersensitivity to paroxetine and excipients, abnormal coagulation parameters, bleeding disorders or conditions which might have predisposed to bleeding
- Individuals with a history of or active suicidal ideation. Assessment included using the Columbia Suicide Severity Rating Scale
- Individuals must not have had a pre-diagnosed mood disorder(s)
- Individuals who were mentally or legally incapacitated
- Individuals supine blood pressure which was persistently higher than 140/90 mmHg at screening
- Individuals with a supine heart rate outside the range 50–90 BPM at screening
- Individuals with a positive screening hepatitis B surface antigen or positive hepatitis C antibody result within 3 months of screening
- Individuals with current or chronic history of liver disease, or known hepatic or biliary abnormalities (except for Gilbert's syndrome or asymptomatic gallstones)
- Individuals with a positive test for human immunodeficiency virus antibody at screening
- Individuals with a positive drug/alcohol screen at screening or prior to dosing
- Individuals with history of regular alcohol consumption within 6 months of the study defined as: an average weekly intake of >21 units for males or >14 units for females
- Individuals who had enrolled into a clinical trial and had received an investigational product within the following time period prior to the first dosing day in the current study: 3 months, five

half-lives, or twice the duration of the biological effect of the investigational product (whichever was longer)

- Individuals who had exposure to >4 new chemical entities within 12 months prior to the first dosing day
- Individuals who used the following medications within 7 days (or 14 days if the drug was a potential enzyme inducer) or five half-lives (whichever was longer) prior to the first dose of the study intervention: monoamine oxidase inhibitors (including linezolid), thioridazine, pimozide, serotonergic drugs (including L-tryptophan, triptans, tramadol, selective serotonin reuptake inhibitor, lithium, and fentanyl), tamoxifen, anti-coagulants, clozapine, phenothiazines, tricyclic anti-depressants, acetylsalicylic acid, non-steroidal anti-inflammatory drugs, Cox-2 inhibitors, anti-arrhythmics, quinolone antibiotics, macrolides (including clarithromycin and erythromycin), ketoconazole, and itraconazole
- Individuals who used non-prescription drugs, including vitamins and herbal and dietary supplements (including St John's Wort) within 7 days (or 14 days if the drug was a potential enzyme inducer) or five half-lives (whichever was longer) prior to the first dose of study intervention
- Individuals who were using any medication other than paracetamol (doses ≤2 g/day)
- Individuals who had consumed Seville oranges, pummelos (members of the grapefruit family), or grapefruit juice from 7 days prior to the first dose of study intervention
- Individuals who were pregnant females as determined by positive serum β-human chorionic gonadotropin (β-HCG) test at screening or serum/urine β-HCG prior to dosing
- Female individuals who were lactating
- Individuals who showed unwillingness or inability to follow the procedures outlined in the protocol
- Individuals with unsuitable veins for cannulation and repeat venipuncture

**Table S1. Predicted effect of random variation (i.e., time effect) at each sampling time[a]**

| Sampling time (n/N, 38/38)[b] | ΔQTcF Estimate (SE) | 95% Confidence Interval |
|---|---|---|
| 1.0 h | 0.34 (0.91) | -1.43, 2.12 |
| 2.0 h | -4.38 (0.91) | -6.16, -2.60 |
| 3.0 h | -7.65 (0.91) | -9.44, -5.87 |
| 4.0 h | -6.30 (0.91) | -8.08, -4.52 |
| 4.5 h | -3.60 (0.91) | -5.38, -1.82 |
| 5.0 h | -1.54 (0.91) | -3.32, 0.25 |
| 5.5 h | 1.86 (0.91) | 0.08, 3.64 |
| 6.0 h | 2.76 (0.91) | 0.99, 4.54 |
| 8.0 h | -1.58 (0.91) | -3.36, 0.20 |
| 10.0 h | -6.86 (0.91) | -8.64, -5.08 |
| 12.0 h | -4.53 (0.91) | -6.30, -2.75 |

[a]Further to QTcF model parameter estimates and predicted ΔQTcF presented in Table 2, estimates of the potential effect of random variation over time were derived for each sampling time after dose.
[b] One individual at 40 mg and two individuals at 60 mg observations were excluded from the analysis.
Equation 1: $\Delta QTc_{i,k}=(\theta_0+\eta_{0,i})+(\theta_1+\eta_{1,i})*C_{i,k}+\theta_{2,k}*TIME_{i,k}+\theta_3(QTc_{i,k=0}-\overline{QTc_0})+\varepsilon_{i,k})$.
Δ, change; h, hour; SE, standard error.

**Table S2. Effect of paroxetine on ECG measures**

| Category | n (%) of individuals with ≥1 value meeting threshold | | |
|---|---|---|---|
| | **Treatment 20 mg (n=33)** | **Treatment 40 mg (n=32)** | **Treatment 60 mg (n=31)** |
| ECG mean HR (BPM) | | | |
| HR <40 | 0 | 0 | 0 |
| HR >120 | 0 | 0 | 0 |
| $\Delta_{Rel}$HR >25% | 0 | 0 | 0 |
| PR interval, aggregate (msec) | | | |
| PR >220 | 0 | 0 | 0 |
| $\Delta_{Rel}$PR >25% | 0 | 0 | 0 |
| QRS duration, aggregate (msec) | | | |
| QRS >120c | 0 | 0 | 0 |
| $\Delta_{Rel}$QRS >25% | 0 | 0 | 0 |
| QTcF interval, aggregate (msec) | | | |
| 450<QTcF – ≤480 | 1 (3.0) | 1 (3.1) | 0 |
| 480<QTcF – ≤500 | 0 | 0 | 0 |
| QTcF >500 | 0 | 0 | 0 |
| 30<ΔQTcF – ≤60 | 0 (0.0) | 3 (9.4) | 1 (3.2) |
| ΔQTcF >60 | 0 | 0 | 0 |

Individuals with multiple values meeting threshold in the same category were counted only once in that category.
ΔRel = 100×(value-baseline)/baseline; Δ = Change from baseline; ΔRel = Relative change from baseline; BPM, beats per minute; ECG, electrocardiogram; HR, heart rate; QTcF, QT interval with Fridericia's correction.

**Table S3. Effect of paroxetine on morphological abnormalities**

| Category | n (%) of individuals with ≥1 value meeting threshold | | |
|---|---|---|---|
| | **Treatment 20 mg (n=33)** | **Treatment 40 mg (n=32)** | **Treatment 60 mg (n=31)** |
| Atrioventricular conduction | | | |
| Short PR interval | 0 | 0 | 1 (3.2) |
| Comparison to a prior ECG | | | |
| >20 BPM increase from baseline and rate ≤130 BPM | 0 | 0 | 1 (3.2) |
| Rhythm not otherwise specified | | | |
| Uncertain supraventricular rhythm | 1 (3.0) | 0 | 1 (3.2) |
| Sinus node rhythms and arrhythmias | | | |
| Sinus bradycardia, 40–49 BPM | 1 | 1 (3.1) | 1 (3.2) |
| Sinus tachycardia, 100–130 BPM | 0 | 1 (3.1) | 0 (0.0) |
| Supraventricular arrhythmias | | | |
| Premature atrial complexes | 0 | 1 (3.1) | 1 (3.2) |
| ST segment, T wave, and U wave | | | |
| QTcF increase from baseline, >30 msec and <60 msec | 1 (3.0) | 2 (6.3) | 2 (6.5) |
| T wave abnormality in precordial leads (non-specific) | 1 (3.0) | 0 (0.0) | 0 (0.0) |
| T wave abnormality in standard leads (non-specific) | 1 (3.0) | 1 (3.1) | 1 (3.2) |
| T wave inversion (non-specific, localized in septal leads: V1V2) | 0 (0.0) | 0 (0.0) | 1 (3.2) |

Individuals with multiple abnormalities in the same category were counted only once in that category. BPM, beats per minute; QTcF, QT interval with Fridericia's correction.

**Figure S1. Final model diagnostic plot. Predicted ΔQTcF vs. conditional studentized residuals.**

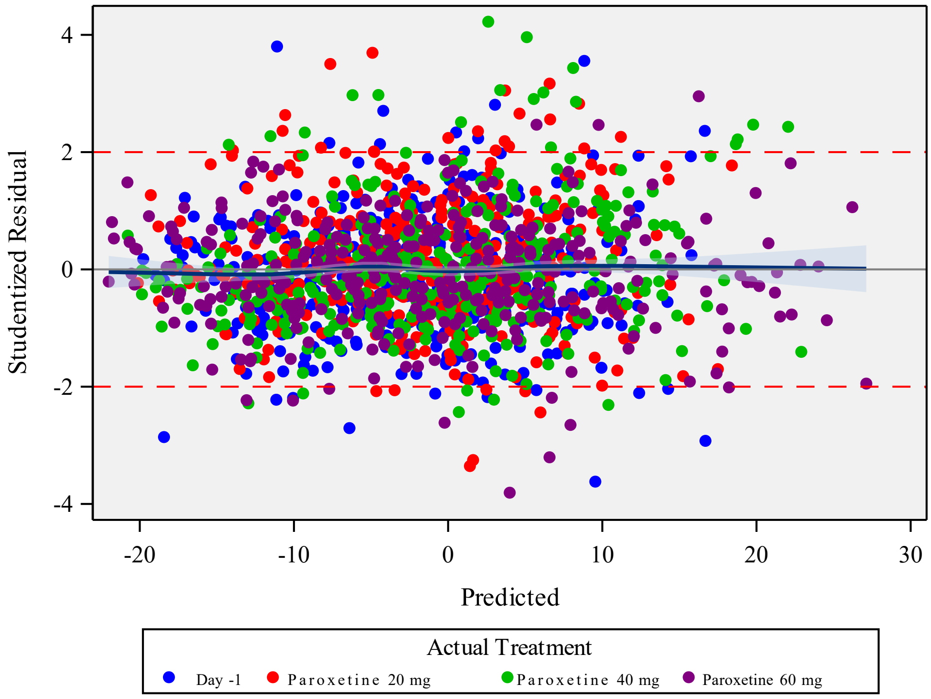

**Figure S2. Final model diagnostic plot. Paroxetine concentrations vs. conditional studentized residuals.**

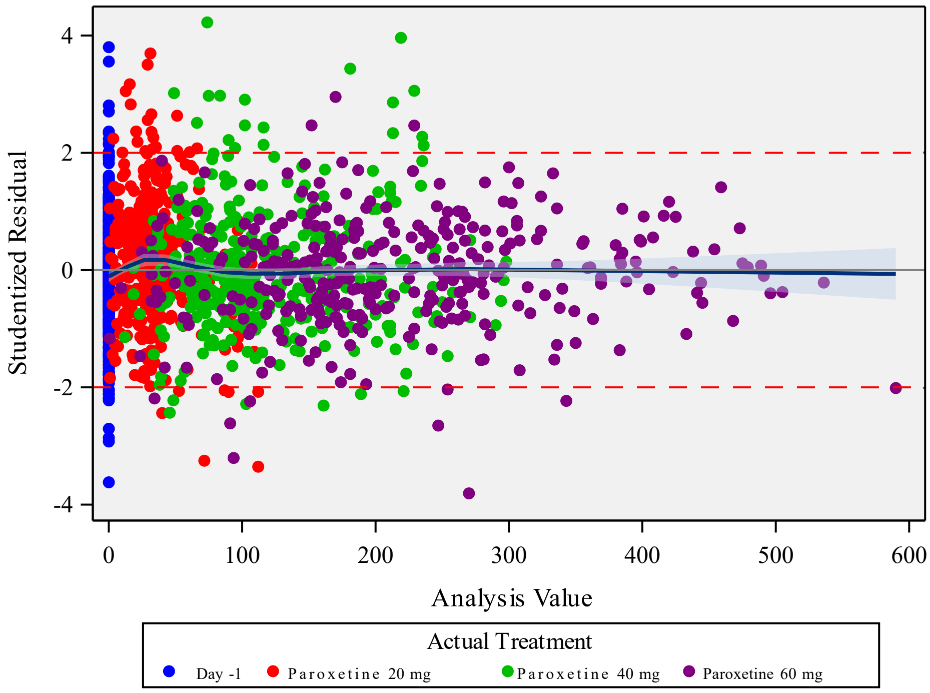

**Figure S3. Final model diagnostic plot. Baseline ΔQTcF vs conditional studentized residuals.**

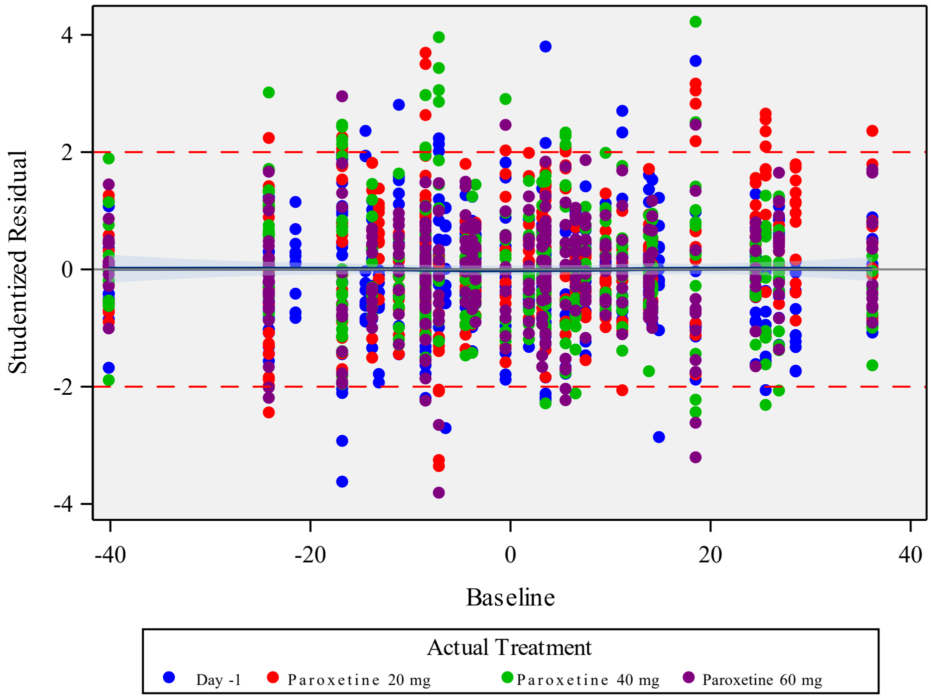

**Figure S4. Final model diagnostic plot. Sampling time after dose vs conditional studentized residuals.**

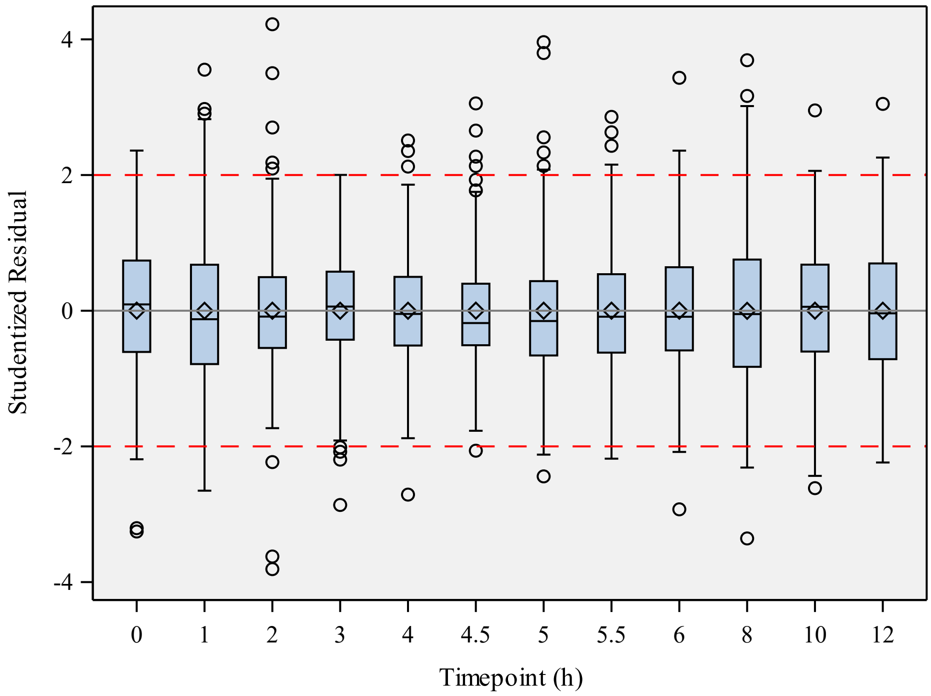


*Boxes represent the 25th and 75th percentiles, the horizontal lines inside the boxes are the median. The squares inside the boxes are the mean values, dots outside the boxes are outliers*

**Figure S5. Final model diagnostic plot. Treatment level vs. conditional studentized residuals**

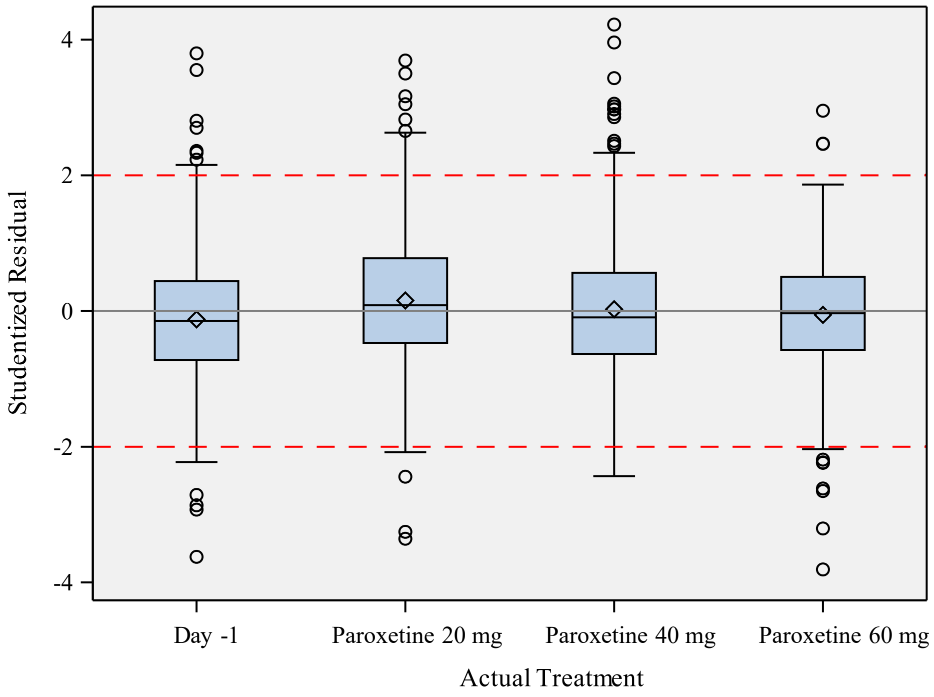


*Boxes represent the 25th and 75th percentiles, the horizontal lines inside the boxes are the median. The squares inside the boxes are the mean values, dots outside the boxes are outliers*

**Figure S6. Final model diagnostic plot. QQ plot of the distribution of conditional studentized residuals.**

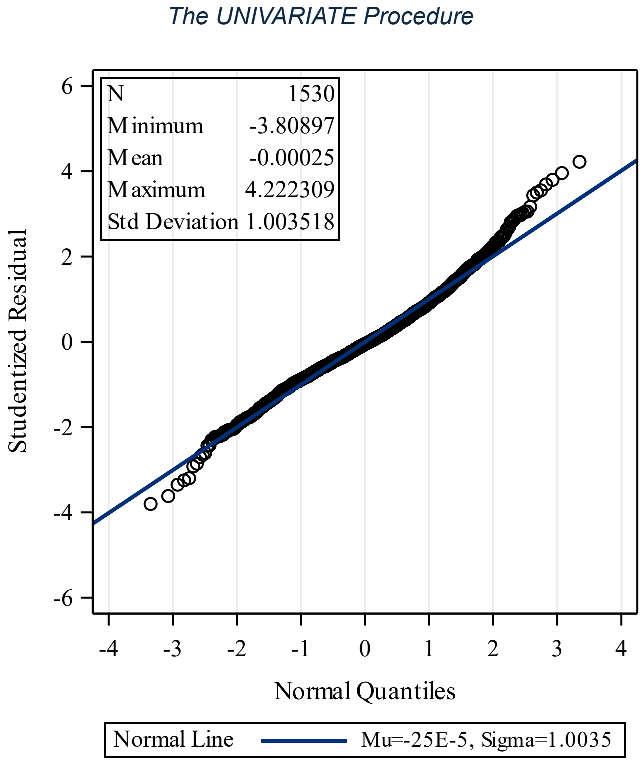

**Figure S7. Final model diagnostic plot. Observed vs conditional predicted ΔQTcF.**

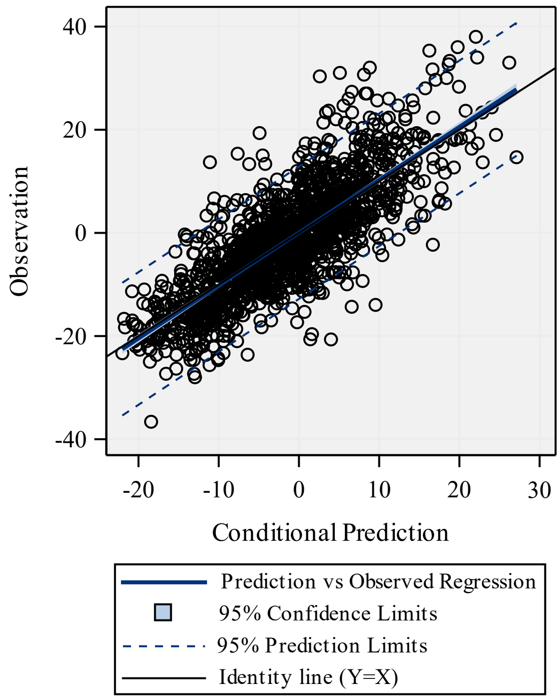

## SAS Code used for the C-QT analysis:

```
proc mixed data=DS(where=(ANL04FL="Y")) noitprint cl noclprint  method=ML plots=none;

title1 j=l "16.1.9.1.2 Primary Endpoint Analysis: Concentrations-QTcF Final Model (PD and PK
Analysis Set) ";

ods output SolutionF=SolF estimates=EstF CovParms=COV Nobs=Nobs Dimensions=Dim;

class USUBJID ATPTN(ref=first) ;

model CHG=BASEC ATPTN PAROX/ddfm=KR solution alphap=.05 cl residual outpm=pred outp=ipred;

random int PAROX/sub=USUBJID type=UN;

estimate "T20" INT 1 PAROX 36.23 /cl alpha=.1 ;

estimate "T40" INT 1 PAROX 128.2 /cl alpha=.1 ;

estimate "T60" INT 1 PAROX 221.4 /cl alpha=.1 ;

footnote1 j=l "Source: Listings 16.2.6.1 and 16.2.9.1";

footnote2 " ";

footnote3 j=l "Program: C-QTcF_lin_&Status..sas ^R'\tab'  Version:&Status ^R'\tab' Run on:
&mydate";

run;
```
